# Supplementary material for: Dietary Intakes of Zinc, Copper, Magnesium, Calcium, Phosphorus, and Sodium by the General Adult Population Aged 20–50 Years in Shiraz, Iran: A Total Diet Study Approach
Source: Nutrients. 2020 Nov 1;12(11):3370. doi: 10.3390/nu12113370 (PMC7693320; doi:10.3390/nu12113370)
Supplement: Supplementary file 1 [file nutrients-12-03370-s001.pdf]

**Supplementary Material**

**Table S1.** The mean concentrations (mg/kg fresh weight) of zinc (Zn), copper (Cu), magnesium (Mg), calcium (Ca), phosphorous (P), and sodium (Na) in foods "as consumed" at food item level in Shiraz, Iran Total Diet Study.

| Food group | Food subgroup | Daily intake (g/d) | %Weight | Zn (mg/kg) | Cu (mg/kg) | Mg (mg/kg) | Ca (mg/kg) | P (mg/kg) | Na (mg/kg) |
|---|---|---|---|---|---|---|---|---|---|
| Bread | Barbari | 20.77 | | 7.58 | 1.46 | 177.78 | 73.79 | 317.53 | 2449.26 |
| | Lavash | 66.15 | | 11.23 | 1.91 | 253.53 | 123.39 | 591.18 | 2458.31 |
| | Sangak | 37.40 | | 9.11 | 1.30 | 274.95 | 145.00 | 483.74 | 968.09 |
| | Taftan | 2.52 | | 10.65 | 2.01 | 286.83 | 166.24 | 173.39 | 2443.94 |
| | **Total** | **126.84** | **4.43** | **9.64** | **1.67** | **248.27** | **127.10** | **391.46** | **2079.90** |
| Cereals and cereal products | Corn, canned | 3.03 | | 1.98 | 0.06 | 45.63 | 11.34 | 145.98 | 1694.46 |
| | Noodle, boiled | 2.08 | | 1.86 | 0.05 | 67.56 | 81.68 | 119.13 | 2005.87 |
| | Pasta, boiled, drained, steamed | 25.19 | | 3.03 | 1.96 | 90.13 | 81.24 | 156.58 | 1585.16 |
| | Rice, white, boiled | 230.67 | | 4.93 | 1.76 | 96.33 | 54.59 | 131.33 | 2076.51 |
| | Rice, white, boiled, drained, steamed | | | 3.63 | 0.36 | 47.18 | 16.84 | 159.18 | 971.76 |
| | Vermicelli, boiled | 2.24 | | 8.13 | 0.36 | 71.88 | 60.44 | 97.03 | 176.21 |
| | **Total** | **263.20** | **9.20** | **3.85** | **0.70** | **69.39** | **54.08** | **132.79** | **1397.17** |
| Legumes | Kidney bean, boiled | 9.77 | | 7.69 | 2.04 | 462.03 | 376.67 | 768.05 | 234.17 |
| | Lentil, boiled | 10.07 | | 12.56 | 2.35 | 1074.25 | 182.90 | 329.75 | 57.73 |
| | Mung bean, boiled | 1.18 | | 13.33 | 4.16 | 415.93 | 150.69 | 948.48 | 138.11 |
| | Soy, saute | 4.90 | | 7.09 | 1.80 | 375.53 | 296.66 | 568.73 | 2272.56 |
| | Split pea, boiled | 5.91 | | 15.43 | 4.76 | 290.13 | 158.79 | 375.78 | 108.46 |
| | **Total** | **31.83** | **1.11** | **11.22** | **3.02** | **523.57** | **233.14** | **598.15** | **562.21** |
| Red meat and meat products | Beef, shank, boiled | 6.26 | | 58.04 | 1.55 | 254.00 | 83.47 | 763.81 | 1654.55 |
| | Beef, shank,fried | | | 73.01 | 1.19 | 306.60 | 95.30 | 1073.52 | 384.12 |
| | Beef, shank,grilled | | | 68.08 | 2.51 | 533.83 | 356.04 | 988.53 | 2422.96 |
| | Lamb, shank,boiled | 17.96 | | 36.21 | 1.25 | 238.85 | 99.23 | 783.72 | 1489.99 |
| | Lamb, shank,fried | | | 65.08 | 2.16 | 231.53 | 51.29 | 1419.43 | 2465.21 |

| | | | | | | | | | |
|---|---|---|---|---|---|---|---|---|---|
| | Lamb, shank,grilled | | | 55.40 | 2.31 | 999.90 | 323.96 | 1038.68 | 2446.01 |
| | Beef hamburger, fried | 3.45 | | 19.42 | 1.42 | 347.97 | 323.12 | 675.71 | 3703.18 |
| | Beef kielbasa | 1.96 | | 5.48 | 0.76 | 91.63 | 91.24 | 643.83 | 2445.91 |
| | Beef sausage, fried | 4.16 | | 9.63 | 2.21 | 137.68 | 187.54 | 1006.43 | 2445.01 |
| | Lamb, kidney, grilled | 3.45 | | 25.22 | 4.87 | 468.18 | 345.49 | 1127.08 | 2430.91 |
| | Lamb, liver, grilled | | | 38.08 | 37.35 | 185.74 | 54.13 | 1469.54 | 1188.63 |
| | Lamb, mince, fried | 6.04 | | 37.03 | 2.06 | 191.63 | 62.74 | 981.63 | 2423.91 |
| | **Total** | **43.28** | **1.51** | **31.68** | **4.46** | **278.68** | **171.55** | **946.92** | **2349.82** |
| Poultry | Chicken, breast, boiled | 29.05 | | 9.79 | 0.60 | 256.42 | 91.74 | 1044.09 | 545.13 |
| | Chicken, breast, fried | | | 7.63 | 2.01 | 248.33 | 73.64 | 1406.98 | 2440.16 |
| | Chicken, breast, grilled | | | 6.18 | 0.76 | 271.26 | 122.70 | 1094.82 | 2665.71 |
| | Turkey, breast, boiled | 1.41 | | 6.48 | 1.31 | 237.13 | 62.04 | 889.73 | 1951.61 |
| | Turkey, breast, fried | | | 23.33 | 1.54 | 903.02 | 770.41 | 1043.10 | 2811.43 |
| | Turkey, breast, grilled | | | 7.63 | 2.01 | 221.93 | 49.84 | 1279.73 | 2443.71 |
| | **Total** | **30.46** | **1.06** | **10.17** | **1.37** | **356.34** | **195.06** | **1126.40** | **2142.96** |
| Fish | Fish, canned | 1.73 | | 5.94 | 0.87 | 149.50 | 76.82 | 527.22 | 2021.75 |
| | Fish, Indo-Pacific king mackerel, grilled | 8.65 | | 5.36 | 1.24 | 343.05 | 143.70 | 1082.09 | 2313.72 |
| | Fish, Indo-Pacific king mackerel, fried | | | 5.38 | 0.71 | 297.80 | 463.22 | 1176.19 | 2962.20 |
| | **Total** | **10.38** | **0.36** | **5.65** | **0.92** | **234.96** | **190.14** | **828.18** | **2329.85** |
| Eggs | Egg, whole, boiled | 39.62 | | 10.47 | 0.46 | 311.65 | 259.61 | 334.90 | 672.49 |
| | Egg, whole, fried | | | 12.19 | 0.40 | 282.38 | 248.15 | 321.11 | 645.47 |
| | **Total** | **39.62** | **1.38** | **11.33** | **0.43** | **297.01** | **253.88** | **328.01** | **658.98** |
| Milk and milk products | Cream cheese, 24-25% milkfat | 3.11 | | 5.33 | 1.06 | 65.48 | 331.44 | 404.08 | 2431.56 |
| | Ultra-filtered feta cheese, 7.5-23% milkfat | 16.86 | | 10.67 | 0.93 | 491.24 | 1520.68 | 618.78 | 4081.93 |
| | Dough (Iranian drinking yogurt), 1.5% milkfat | 47.43 | | 2.23 | 2.06 | 52.78 | 215.89 | 150.63 | 2165.31 |
| | Ice cream | 12.93 | | 3.98 | 2.41 | 114.73 | 394.59 | 345.18 | 372.66 |
| | Cocoa milk, 1.5-2.4% milkfat, fluid | 13.46 | | 3.36 | 1.10 | 426.31 | 487.27 | 163.71 | 121.70 |
| | Fruit-milk, 1.5-3% milkfat, fluid | | | 1.75 | 1.05 | 285.77 | 424.37 | 120.49 | 155.20 |

| | | | | | | | | | |
|---|---|---|---|---|---|---|---|---|---|
| | Milk, low-fat, 0.6-1.5% milkfat, fluid | 60.26 | | 2.09 | 0.80 | 316.57 | 478.28 | 131.53 | 154.56 |
| | Milk, whole, 3% milkfat, fluid | 34.81 | | 6.73 | 1.11 | 82.18 | 398.09 | 251.33 | 344.21 |
| | Yogurt, low-fat, 1-1.5% milkfat | 29.01 | | 3.31 | 0.68 | 360.04 | 633.17 | 188.50 | 206.26 |
| | Yogurt, medium-fat, 1.5-2% milkfat | 20.46 | | 3.93 | 1.19 | 447.98 | 772.56 | 161.65 | 259.49 |
| | Yogurt, full-fat, 4-5% milkfat | 34.66 | | 3.33 | 1.31 | 74.48 | 342.54 | 316.03 | 589.16 |
| | Yogurt, Greek, 7% milkfat | 1.37 | | 4.48 | 0.96 | 80.68 | 434.69 | 404.38 | 1426.26 |
| | **Total** | **274.36** | **9.59** | **4.42** | **1.24** | **222.02** | **543.43** | **283.11** | **1106.35** |
| Raw vegetables | Carrot | 14.71 | | 3.03 | 1.36 | 57.28 | 214.54 | 66.63 | 286.36 |
| | Cucumber, peeled | 63.72 | | 3.03 | 1.36 | 57.28 | 214.54 | 66.63 | 286.36 |
| | Finger hot pepper | 3.97 | | 1.53 | 1.91 | 165.63 | 66.29 | 226.18 | 23.86 |
| | Fresh herb platter | 11.51 | | 4.58 | 1.91 | 485.98 | 542.69 | 143.98 | 1232.51 |
| | Green bell pepper | 12.38 | | 0.48 | 0.75 | 82.12 | 70.23 | 56.59 | 60.23 |
| | Green cabbage | 12.05 | | 2.50 | 1.14 | 387.78 | 371.66 | 77.58 | 743.51 |
| | Lettuce | 26.36 | | 2.38 | 2.06 | 103.93 | 156.34 | 111.48 | 79.06 |
| | Onion | 33.72 | | 1.68 | 1.76 | 81.23 | 126.74 | 110.18 | 287.65 |
| | Tomato | 105.44 | | 1.14 | 1.00 | 342.14 | 142.17 | 41.88 | 14.94 |
| | **Total** | **283.86** | **9.92** | **2.13** | **1.48** | **236.00** | **214.32** | **98.59** | **306.70** |
| Cooked vegetables | Celery, saute | 6.60 | | 2.33 | 1.36 | 211.08 | 419.09 | 63.73 | 1806.61 |
| | Eggplant, fried | 4.06 | | 0.88 | 1.19 | 456.98 | 123.85 | 45.80 | 11.93 |
| | Fines herbs, fried | 14.15 | | 4.59 | 1.71 | 446.13 | 526.86 | 226.58 | 481.95 |
| | Green cabbage, saute | 2.64 | | 0.33 | 0.66 | 88.73 | 110.89 | 84.93 | 65.26 |
| | Mushroom, fried | 7.07 | | 5.23 | 4.21 | 74.28 | 55.24 | 229.63 | 89.41 |
| | Onion, fried | 21.41 | | 2.32 | 1.37 | 466.32 | 212.25 | 69.23 | 3.09 |
| | Spinach, saute | 4.25 | | 5.84 | 2.17 | 764.37 | 532.66 | 229.38 | 595.77 |
| | Squash, saute | 3.07 | | 4.78 | 1.96 | 121.63 | 119.74 | 179.13 | 253.10 |
| | Turnip, boiled | 7.45 | | 1.95 | 1.41 | 102.98 | 152.04 | 46.33 | 604.56 |
| | **Total** | **70.69** | **2.47** | **3.14** | **1.78** | **303.61** | **250.29** | **130.52** | **434.63** |
| Potatoes | Potato, peeled, sliced, fried | 10.77 | | 3.38 | 2.11 | 222.13 | 60.94 | 254.63 | 1384.56 |
| | Potato, boiled, peeled | 18.14 | | 3.28 | 1.81 | 162.58 | 60.94 | 99.78 | 118.51 |
| | **Total** | **28.91** | **1.01** | **3.33** | **1.96** | **192.35** | **60.94** | **177.20** | **751.54** |

| | | | | | | | | | |
|---|---|---|---|---|---|---|---|---|---|
| Fruits | Apple, peeled | 70.23 | | 1.70 | 1.76 | 42.38 | 38.69 | 21.98 | 266.65 |
| | Apricot | 2.80 | | 0.58 | 1.56 | 61.83 | 28.94 | 63.13 | 203.90 |
| | Banana | 20.40 | | 2.88 | 1.71 | 152.18 | 45.09 | 42.58 | 236.45 |
| | Cantaloupe | 50.07 | | 1.64 | 1.04 | 242.59 | 96.20 | 35.08 | 187.93 |
| | Cherries, sweet | 1.86 | | 0.13 | 1.71 | 76.18 | 69.14 | 106.23 | 260.65 |
| | Date | 24.57 | | 3.23 | 3.61 | 246.18 | 188.84 | 239.48 | 39.56 |
| | Fig | 1.38 | | 0.68 | 1.76 | 175.48 | 184.94 | 28.83 | 265.50 |
| | Grapefruit | 3.35 | | 0.03 | 1.21 | 71.33 | 107.34 | 84.58 | 219.10 |
| | Grapes | 28.55 | | 0.86 | 1.69 | 73.55 | 44.64 | 66.45 | 214.45 |
| | Kiwi, peeled | 5.26 | | 1.68 | 2.21 | 88.73 | 121.69 | 64.18 | 273.00 |
| | Lemon | 4.13 | | 1.92 | 1.01 | 275.43 | 199.01 | 34.42 | 164.16 |
| | Melon | 29.19 | | 2.28 | 1.79 | 85.65 | 69.46 | 12.35 | 200.26 |
| | Nectarine | 7.04 | | 1.60 | 1.76 | 72.38 | 32.69 | 26.73 | 263.55 |
| | Orange | 104.02 | | 1.18 | 1.16 | 67.03 | 121.34 | 55.13 | 265.35 |
| | Peach, with skin | 22.41 | | 1.28 | 1.61 | 59.83 | 33.54 | 56.48 | 251.95 |
| | Pear, with skin | 2.37 | | 0.43 | 1.56 | 42.73 | 37.69 | 33.23 | 213.65 |
| | Pineapple | 0.25 | | 0.03 | 2.11 | 90.08 | 55.04 | 16.33 | 3.76 |
| | Pineapple, canned, solids | 1.53 | | 0.21 | 1.02 | 81.73 | 56.90 | 7.23 | 232.13 |
| | Plum, damson, with skin | 6.44 | | 3.03 | 1.41 | 58.28 | 47.89 | 18.58 | 23.47 |
| | Plum, mirabelle, with skin | | | 1.50 | 1.16 | 47.68 | 29.34 | 10.93 | 1.05 |
| | Pomegranate | 35.86 | | 1.97 | 1.42 | 396.09 | 127.55 | 61.81 | 127.10 |
| | Sweet lemon | 23.86 | | 0.93 | 1.06 | 54.03 | 101.84 | 40.13 | 214.50 |
| | Tangerine | 22.65 | | 1.08 | 1.61 | 68.08 | 185.94 | 64.98 | 248.75 |
| | Watermelon | 55.28 | | 0.88 | 1.31 | 65.63 | 67.24 | 48.38 | 244.30 |
| | **Total** | **523.48** | **18.30** | **1.28** | **1.61** | **114.87** | **89.23** | **53.23** | **200.39** |
| Fruit juices and soft beverages | Apple juice, homemade unsweetened | 1.12 | | 0.25 | 0.69 | 43.18 | 90.48 | 14.30 | 146.06 |
| | Cantaloupe juice, homemade, unsweetened | 3.25 | | 2.71 | 0.75 | 686.66 | 737.34 | 16.34 | 459.93 |
| | Carrot juice, homemade, unsweetened | 7.38 | | 1.02 | 1.14 | 146.21 | 115.94 | 108.76 | 191.98 |

| | | | | | | | | | |
|---|---|---|---|---|---|---|---|---|---|
| | Orange juice, homemade, unsweetened | 4.18 | | 1.46 | 0.71 | 77.14 | 90.85 | 60.13 | 142.21 |
| | Industrial Juice | 14.92 | | 0.88 | 0.50 | 8.03 | 6.09 | 9.03 | 166.91 |
| | **Total** | **30.85** | **1.08** | **1.26** | **0.76** | **192.24** | **208.14** | **41.71** | **221.42** |
| Nuts and dried fruits | Apricot, dried slices | 1.27 | | 3.58 | 4.56 | 274.43 | 291.09 | 400.08 | 143.86 |
| | Fig, dried | 2.13 | | 4.81 | 1.56 | 565.51 | 1292.95 | 254.95 | 251.63 |
| | Raisins | 2.71 | | 1.18 | 4.11 | 173.23 | 189.24 | 332.48 | 787.91 |
| | Sunflower seeds | 2.62 | | 38.18 | 11.35 | 4465.23 | 7.26 | 2187.86 | 1642.49 |
| | Walnuts | 3.11 | | 22.58 | 12.91 | 773.88 | 247.89 | 891.68 | 123.16 |
| | **Total** | **11.84** | **0.41** | **14.06** | **6.90** | **1250.45** | **405.68** | **813.41** | **589.81** |
| Cookies, cakes, and pastry | Biscuit, plain Sweet | 12.36 | | 6.36 | 3.23 | 168.03 | 83.99 | 221.35 | 1124.34 |
| | Cake, sponge, frosted | 3.88 | | 5.68 | 1.96 | 107.48 | 246.44 | 355.48 | 357.26 |
| | Cake, sponge, plain | 7.36 | | 4.43 | 0.51 | 116.29 | 171.11 | 800.83 | 1679.47 |
| | Cookie, plain | | | 6.55 | 0.60 | 331.11 | 122.11 | 187.17 | 468.83 |
| | Danish pastry | 3.42 | | 4.96 | 1.80 | 82.68 | 89.28 | 174.35 | 650.76 |
| | Popover | 1.13 | | 5.78 | 1.01 | 106.03 | 84.74 | 231.73 | 2178.56 |
| | Puff pastry | 1.17 | | 4.03 | 2.26 | 113.18 | 119.69 | 255.58 | 825.01 |
| | **Total** | **29.32** | **1.02** | **5.38** | **1.80** | **133.51** | **128.46** | **288.75** | **1035.02** |
| Snacks | Cheese puffs | **3.28** | **0.11** | **4.63** | **0.41** | **137.38** | **218.64** | **341.48** | **2444.66** |
| Fats and oils | Butter | 1.93 | | 1.34 | 0.78 | 7.79 | 67.72 | 57.50 | 7.05 |
| | Cream | 2.22 | | 1.33 | 0.50 | 37.28 | 178.59 | 235.18 | 308.31 |
| | Oil, cooking | 8.29 | | 0.03 | 0.50 | 5.20 | 25.94 | 1.43 | 121.01 |
| | Oil, frying | 6.06 | | 3.18 | 0.45 | 6.95 | 30.79 | 26.80 | 241.61 |
| | Oil, hydrogenated | 6.04 | | 1.61 | 0.77 | 6.60 | 44.07 | 34.24 | 216.49 |
| | **Total** | **24.54** | **0.86** | **1.50** | **0.60** | **12.76** | **69.42** | **71.03** | **178.90** |
| Honey and sugar | Honey | 5.09 | | 0.58 | 0.50 | 13.53 | 25.94 | 23.23 | 172.81 |
| | Rock candy | 2.32 | | 1.80 | 0.82 | 1.75 | 25.05 | 9.49 | 191.90 |
| | White Sugar | 7.02 | | 0.23 | 0.40 | 1.63 | 22.14 | 4.38 | 131.71 |
| | **Total** | **14.43** | **0.50** | **0.87** | **0.57** | **5.63** | **24.38** | **12.36** | **165.48** |

| | | | | | | | | | |
|---|---|---|---|---|---|---|---|---|---|
| Condiments | Mayonnaise | 3.46 | | 3.63 | 1.26 | 37.98 | 165.99 | 41.18 | 2446.21 |
| | Pickled cucumber | 8.92 | | 1.28 | 0.11 | 54.88 | 48.54 | 42.93 | 2447.51 |
| | Pickles | 13.57 | | 1.53 | 1.02 | 45.15 | 105.80 | 35.16 | 2420.17 |
| | Table and cooking salt | 5.23 | | 1.75 | 0.81 | 23.48 | 99.84 | 22.65 | 214836.25 |
| | Tomato Paste | 21.04 | | 5.08 | 3.11 | 242.18 | 145.39 | 307.08 | 2421.86 |
| | Lemon Juice | 17.39 | | 0.68 | 1.31 | 56.03 | 83.34 | 34.08 | 400.71 |
| | Unripe-grape verjuice | | | 1.95 | 0.45 | 37.38 | 22.89 | 17.78 | 202.96 |
| | Vinegar | | | 0.45 | 0.73 | 38.52 | 67.11 | 37.54 | 78.08 |
| | **Total** | **69.60** | **2.43** | **2.38** | **1.19** | **74.61** | **103.89** | **79.80** | **37466.54** |
| Drinking water | Tap water | **950.46** | **33.22** | **4.58** | **1.06** | **39.83** | **203.19** | **27.70** | **264.30** |
| **Total diet** | - | **2861.23** | **100** | - | - | - | - | - | - |
